# Effect of intravenous low-dose norepinephrine on blood loss in non-tourniquet total knee arthroplasty under general anesthesia: a randomized, double-blind, controlled, single-center trial

**DOI:** 10.1186/s13018-023-04360-w

**Published:** 2023-12-07

**Authors:** Shijie Chen, Fenqi Luo, Yuan Lin, Guoyu Yu, Jun Luo, Jie Xu

**Affiliations:** 1 Shengli Clinical College of Fujian Medical University, No. 134 East Street, Fuzhou, Fujian China; 2 Department of Orthopedic, Fujian Provincial Hospital, No. 134 East Street, Fuzhou, Fujian China

**Keywords:** Total knee replacement, Low-dose epinephrine, Intravenous pump infusion, Intraoperative blood loss

## Abstract

**Objective:**

This prospective trial aimed to evaluate the effects of low-dose intravenous norepinephrine (NE) on intraoperative blood loss and bleeding from osteotomy sites during non-tourniquet total knee arthroplasty (TKA) under general anesthesia.

**Methods:**

A total of 120 patients who underwent TKA between December 2020 and May 2022 were enrolled and randomly assigned to the intravenous low-dose NE Group (NE Group) or the control group (C Group). During surgery, NE Group received 0.05–0.1 μg/(kg min) of NE intravenously to raise and maintain the patient's mean arterial pressure (MAP). C Group received the same dose of saline as placebo. Intraoperative blood loss, bleeding score at osteotomy sites, Δlactate levels (Lac), postoperative complications, and transfusion rate during hospitalization were compared between groups.

**Results:**

Intraoperative and osteotomy blood loss was significantly lower in the NE Group than in the C Group (*P* < 0.001). No significant difference was observed in ΔLac between groups (*P* > 0.05). There was no significant difference in complications between the groups 3 days after surgery (*P* > 0.05). In addition, there was no significant difference in blood transfusion rates between the two groups during hospitalization (*P* > 0.05).

**Conclusion:**

In non-tourniquet TKA under general anesthesia, low-dose intravenous NE safely and effectively reduced intraoperative blood loss and provided a satisfactory osteotomy site while maintaining a higher MAP.

## Introduction

The management of intraoperative blood loss in total knee arthroplasty (TKA) has always been a focus of attention for orthopedic surgeons. The use of tourniquets can significantly reduce the intraoperative blood loss and operative time during TKA [1–3]. However, the use of tourniquets may increase the rate of deep venous thrombosis and may be associated with ischemia–reperfusion injury, limb swelling and pain, and reduced muscle strength, which are detrimental to early postoperative recovery [4–10]. The use of tourniquets remains controversial in TKA [11]. Controlled hypotension is commonly used for hemostasis in TKA without tourniquets. Controlled hypotension reduces the amount of blood in the surgical field by reducing peripheral vascular perfusion pressure [12, 13]. However, increasing evidence suggests that controlled hypotension can decrease systemic vascular resistance and blood pressure, which can lead to acute renal dysfunction [12, 14–16]. Tranexamic acid is a synthetic anti-fibrinolytic drug that inhibits plasmin degradation of fibrinogen to exert a hemostatic effect [17, 18]. Tranexamic acid is widely administered via intravenous infusion in TKA without tourniquets or controlled hypotension. However, a meta-analysis showed that although intravenous tranexamic acid can significantly reduce perioperative and postoperative blood loss in TKA, it cannot reduce intraoperative blood loss or guarantee a clear bone-cut surface. This limitation significantly hampers its clinical utility [19]. Therefore, reducing intraoperative blood loss, providing a clear bone-cut surface without using tourniquets, and ensuring that blood pressure does not drop excessively have become new focus areas for research.

Norepinephrine (NE) has potent α-adrenergic receptor activation activity, which can stimulate α-1 adrenergic receptors on peripheral vascular smooth muscles [20]. Researchers generally believe that NE can reduce bleeding by constricting small and medium arteries and pre-capillary sphincters, increasing pre-capillary resistance, reducing capillary perfusion pressure, and reducing peripheral blood flow [21, 22]. In addition, studies have found that intravenous infusion of NE can reduce peripheral vascular tension caused by general anesthesia, contract resistant vessels, force blood stagnation in the periphery to re-enter circulation, promote blood redistribution, effectively increase blood pressure, and ensure hemodynamic safety [23–25]. However, the effect of intravenous infusion of low-dose NE on intraoperative blood loss, bone-cut surface oozing, and related organ perfusion indicators during TKA without tourniquets under general anesthesia has not been reported.

## Patients and methods

### Study design

This randomized, controlled, double-blind, single-center trial was conducted between December 2020 and May 2022 at the Department of Orthopedics of Fujian Provincial Hospital, Fuzhou, China. This trial was conducted in accordance with the Declaration of Helsinki and approved and supervised by the Fujian Provincial Hospital Ethics Committee (K2020-09–075). The trial protocol was registered in the Chinese Clinical Trial Registry (ChiCTR2000040311). This study was conducted according to the Basic & Clinical Pharmacology & Toxicology policy for experimental and clinical studies [26].

### Patients

Patients scheduled for an initial unilateral TKA were screened. All patients were ≥ 18 years old and provided written informed consent. The selection criteria were as follows: (1) patients’ clinical symptoms, signs, and radiological examination findings were consistent with the diagnosis of osteoarthritis; (2) patients’ knee joint Kellgren-Lawrence grading was III–IV and the American Society of Anesthesiologists (ASA) physical status grading was II-III; and (3) patients had normal routine preoperative blood examination and coagulation function. The exclusion criteria were as follows: (1) hemorrhagic or thrombotic diseases; (2) current anticoagulation therapy; (3) allergic reactions to drugs involved in this trial; and (4) severe anemia, acute myocardial infarction, stroke, severe liver or kidney disease, or other conditions that are not suitable for surgery.

### Randomization and blinding

Patients were randomly divided into an intravenous low-dose NE group (NE Group) and a control group (C Group) using a computer-generated randomization list with a block size of 30 and a 1:1 allocation ratio. The patients were registered by a dedicated investigator and assigned sealed, unique, randomly numbered, opaque envelopes in which the allocation was concealed. Before surgery, an anesthesiologist who did not perform the surgery prepared the trial drugs (low-dose NE or saline solution) according to the allocation noted in the envelope. The remaining investigators, surgeons, anesthesiologists, and patients were blinded to allocation.

### Anesthesia

Both groups underwent intravenous-inhalation combined general anesthesia performed by the same team of anesthesiologists [27]. Radial artery cannulation and peripheral intravenous catheter placement were performed to measure invasive blood pressure and intravenous infusion. Anesthesia induction was conducted using intravenous midazolam 0.03 mg/kg, sufentanil 0.4 μg/kg, etomidate 0.3 mg/kg, and cisatracurium 0.15 mg/kg. Mechanical ventilation was performed after tracheal intubation with a tidal volume of 8 ml/kg, respiratory rate adjusted to maintain end-tidal carbon dioxide pressure within the range of 35–45 mmHg, inspiratory-to-expiratory ratio of 1:2, and inhalation of 60% oxygen at a flow rate of 2 L/min. After anesthesia induction, propofol 2–3 mg/(kg·h), remifentanil 0.1–0.3 μg/(kg·min), and inhaled desflurane 1%-2% were used to maintain anesthesia. During surgery, bispectral index was maintained between 40–60, and patient temperature was maintained above 36℃. Both groups received standardized fluid infusion, with an intravenous crystalloid mixed solution (1:1 ratio of hydroxyethyl starch injection and sodium, potassium, magnesium, and calcium glucose injection) infused at a rate of 0.1–0.3 ml/(kg·min) after anesthesia induction to maintain mean arterial pressure between 65–75 mmHg.

### Trial intervention

NE Group: From 10 min after anesthesia induction to the end of surgery, a micro-injection pump was utilized to intravenously inject norepinephrine at a rate of 0.05–0.1 mg/(kg·min), which raises and stabilizes the mean arterial pressure at 80–90 mmHg. A NE solution was prepared by diluting 2 mg NE in 50 ml of physiological saline.

C Group: From 10 min after anesthesia induction to the end of surgery, an intravenous injection of the same amount of physiological saline as the placebo was administered through a pump, and additional adjustments to blood pressure were not made during the operation.

### Surgery

TKAs were performed by the same senior surgeon (JX) in both groups following a standardized procedure. The medial femoral muscle approach was used to expose the articular cavity [28]. Both groups underwent resection of the hypertrophic synovium and infrapatellar fat pad, followed by subperiosteal release of the synovial capsule to facilitate lateral patellar displacement. Peripheral osteophytes were removed and routine medial release was performed, with lateral release performed when necessary. The anterior and posterior cruciate ligaments as well as the medial and lateral menisci were removed, and soft tissue balance was achieved. The soft tissue, lateral collateral ligaments, and posterior joint capsule were appropriately released to ensure equal mediolateral gaps in extension and flexion. Femoral osteotomy was performed using an intramedullary positioning system with 5–7° valgus. The tibia was positioned using an extramedullary positioning system with 3°–5° varus. After the test piece was deemed suitable, an ATTUNE prosthesis (Johnson & Johnson Co., USA) was installed and fixed with bone cement. The patellar joint surface was trimmed using a swing saw, the patella was contoured, and the patellar edge denervated. Before closure of the joint capsule, an analgesic (200 mg of ropivacaine and 0.5 mg of norepinephrine diluted in 100 mL of saline solution) was periarticularly infiltrated. Tourniquets or drainage tubes were not used. Routine prophylactic intravenous cefuroxime (1.5 g) was administered 30 min before surgery and at 12-h intervals. For ethical reasons, both groups of patients received 15 mg/kg intravenous tranexamic acid before surgery and 3 h after surgery [29].

### Perioperative management

A standardized transfusion protocol was followed during hospitalization:2U (400 ml) of erythrocyte suspension was transfused if the hemoglobin level was < 70 g/l. Postoperative analgesic regimens were as follows: (1) a daily oral dose of 400 mg of celecoxib was administered from postoperative day 1 (POD1) to discharge; and (2) if the patient developed pain, 50 mg of flurbiprofen was administered intravenously by the ward physician according to the pain presentation. Thromboprophylaxis consisted of the following: (1) 4000 AXaIU of enoxaparin was administered subcutaneously daily, and intermittent pneumatic compression (IPC) was performed from POD1 to discharge; (2) a daily oral dose of 10 mg of rivaroxaban was administered for 28 days after discharge.

### Outcome measures

Age, gender, body mass index (BMI), ASA classification, Kellgren-Lawrence classification, red blood cell count (RBC), hemoglobin concentration (Hb), hematocrit (Hct), platelet count (PLT), prothrombin time (PT), activated partial thromboplastin time (APTT), D-dimer, and chronic diseases were recorded to evaluate comparability between groups. Surgical time, intraoperative fluid volume, and intraoperative urine output were recorded as general intraoperative conditions.

Vein blood collection for hemoglobin concentration testing was performed upon completion of surgery. The estimated blood volume was calculated using Nadler formula (Formula 1) [30]. The intraoperative blood loss was calculated by entering the pre and postoperative hemoglobin concentrations and estimated blood volume into the hemoglobin balance formula (Formulas 2 and 3).

1 $$EBV = k_{1} \times H^{3} + k_{2} \times W + k_{3} *$$

2 $$Hb_{l} = EBV \times \left( {Hb_{pre} - Hb_{post} } \right) \times 0.001$$

3 $$V_{l} = 1000 \times \frac{{Hb_{l} }}{{Hb_{pre} }}$$

*Men: k_1_ = 0.3669, k_2_ = 0.03219, k_3_ = 0.6041; Women: k_1_ = 0.3561, k_2_ = 0.03308, k_3_ = 0.1833.

Note: EBV (ml), estimated total blood volume. H (m): height. W (kg): weight. HbL (g): total hemoglobin loss Hbpre (g/L): preoperative hemoglobin concentration. Hbpost (g/L): postoperative hemoglobin concentration. VL (ml): total blood loss.

During surgery, the bleeding condition of the bone-cutting surface was evaluated and graded by the same senior surgeon using the bleeding score method for grading the distal femur bone-cutting surface [31, 32] as follows:1 for minimal bleeding, 2 for mild bleeding that does not affect the identification of anatomical structures, 3 for moderate bleeding that slightly affects the identification of anatomical structures but does not require a pause in the surgery, and 4 for severe bleeding that significantly affects the identification of anatomical structures and requires cleaning before continuing the surgery.

Lactate levels (Lac) in arterial blood were measured before and after low-dose NE administration, and the difference (ΔLac) was recorded to assess the perfusion of the patient's systemic organs. All patients were followed up for 3 days after surgery and underwent lower-limb venous Doppler ultrasonography to monitor complications. Transfusion records during hospitalization were documented for both groups of patients.

All measurements were performed by an investigator at the indicated time intervals and analyzed by the Clinical Biochemical Department of Fujian Provincial Hospital.

### Statistical analysis

This study was conducted as a superiority trial. Our pre-experimental data showed that the mean intraoperative blood loss and standard deviation (SD) was 492.62 ± 135.24 ml in the C Group and 326.46 ± 96.28 ml in the NE Group. Assuming a one-sided α level of 0.05, a power of 0.9, a difference in means of 50 ml, and a 10% loss to follow-up, a minimum of 60 patients was required in each group.

All data were assessed for normality using histograms, quantile plots (Q-Q plots), and the Kolmogorov–Smirnov test before analysis. Normally distributed continuous data were presented as means and SD; otherwise, they were presented as medians and interquartile ranges (IQR). Categorical data were presented as frequencies and percentages. Student's t-test was used to compare normally distributed data, while the Mann–Whitney U test was used to compare skewed distributed data. The Fisher’s test or chi-square test was used to compare categorical data. Group comparisons for intraoperative blood loss and bone-cutting surface bleeding scores were one-sided, whereas all other data comparisons were two-sided. All statistical analyzes were performed prior to the blinded analysis. All data were analyzed using SPSS software (version 23.0; IBM Corp., Armonk, NY, USA). Statistical significance was set at *P* < 0.05.

## Results

### Demographic baseline characteristics

**Fig. 1:**
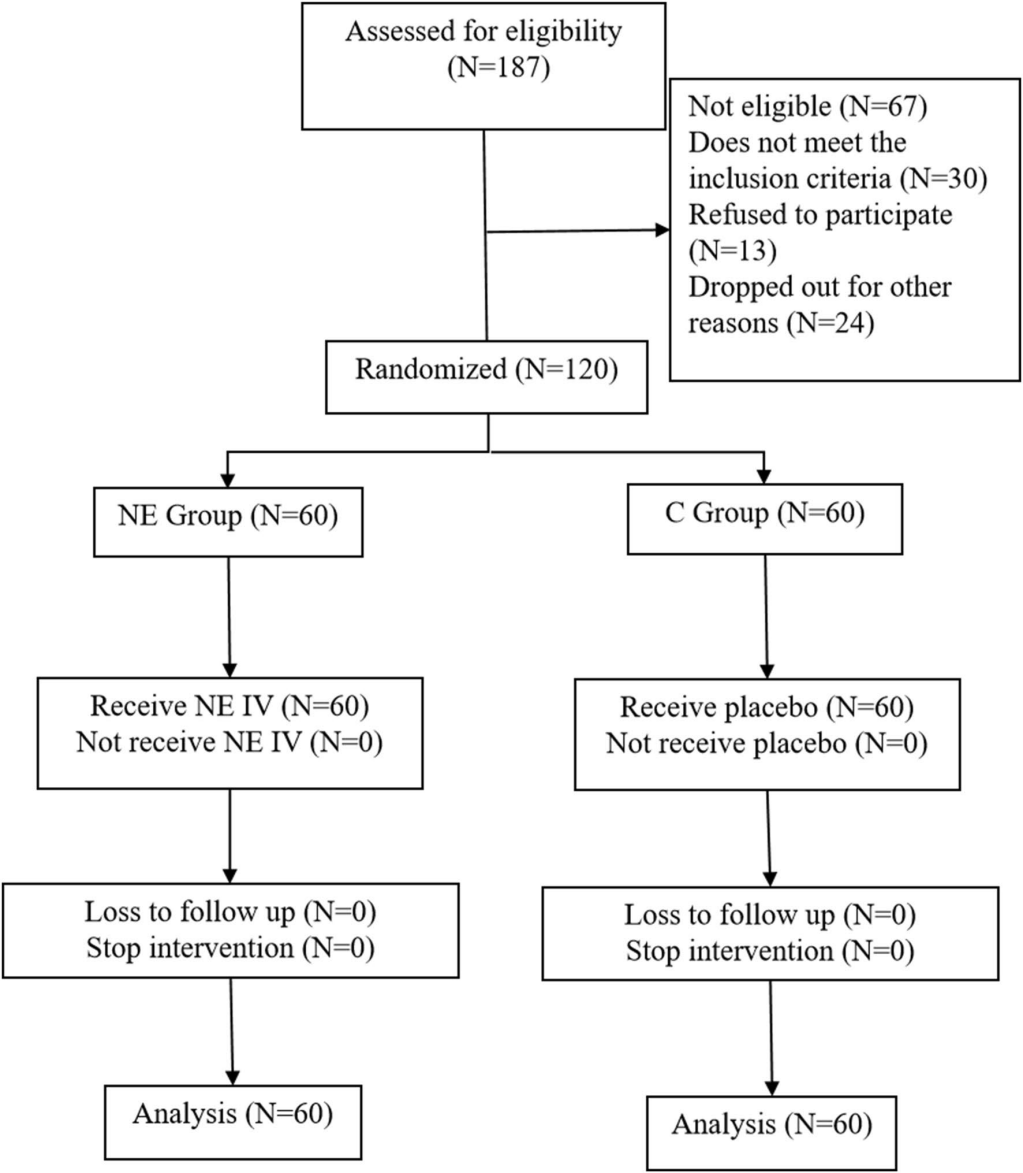
Flowchart of recruiting participants and analysis

A total of 187 patients were screened for eligibility; 30 did not meet the inclusion criteria, 13 refused to participate, and 24 changed their surgical schedules. Finally, 120 patients were randomly enrolled. Data for all patients were available, and no patients were excluded from the trial (Fig. 1). Experimental data from all 120 patients were available. There were 12 male and 48 female patients in the NE group, and 14 male and 46 female patients in the C group. Baseline characteristics, including age, sex, BMI, ASA grade, K-L grade, preoperative blood tests, and chronic diseases, were similar between the groups (all *P* > 0.05) (Table 1).

**Table 1:** Baseline demographic characteristics

	NE group (n = 60)	C group (n = 60)	*t*/*χ*^2^/*Z*	*P* value
Age (yr)	68.30 ± 6.65	67.63 ± 7.14	− 0.530	0.597*
*Gender*			0.196	0.658‡
Male	12 (20.0%)	14 (23.3%)		
Female	48 (80.0%)	46 (76.7%)		
*BMI* (kg/m^2^)	25.50 ± 3.11	26.41 ± 3.51	1.513	0.133*
*ASA grade*			0.159	0.690‡
II	19 (31.7%)	17 (28.3%)		
III	41 (68.3%)	43 (71.7%)		
*K-L grade*			0.051	0.822‡
III	48 (80.0%)	47 (78.3%)		
IV	12 (20.0%)	13 (21.7%)		
*Preoperative blood detection*				
RBC (× 10^9^/L)	4.49 ± 0.52	4.53 ± 0.45	0.429	0.669*
Hb (g/L)	133.03 ± 11.31	136.32 ± 13.00	1.476	0.143*
Hct	0.41 ± 0.03	0.42 ± 0.03	1.646	0.102*
PLT (× 10^9^/L)	240.30 ± 54.52	246.08 ± 55.96	0.573	0.567*
PT (s)	10.66 ± 0.71	10.69 ± 0.72	0.243	0.809*
APTT (s)	25.65 ± 2.21	25.68 ± 1.78	0.073	0.942*
D-D (mg/L)	0.35 (0.25, 0.75)	0.49 (0.26, 0.91)	− 1.449	0.147†
*Chronic diseases*				
*Osteoporosis*			0.086	0.769‡
Yes	6 (10.0%)	7 (11.7%)		
No	54 (90.0%)	53 (88.3%)		
*Hypertension*			0.304	0.581‡
Yes	35 (58.3%)	32 (53.3%)		
No	25 (44.2%)	28 (46.7%)		
Preoperative MAP (mmHg)	93.83 (89.33, 98.83)	94.54 ± 9.79	− 0.068	0.946†
*Diabetes*			0.164	0.685‡
Yes	18 (30.0%)	16 (26.7%)		
No	42 (70.0%)	44 (73.3%)		
Preoperative blood sugar (mmol/L)	5.39 (5.01, 6.38)	5.67 (5.20, 6.33)	− 0.908	0.364†

BMI, Body Mass Index, ASA, American Society of Anesthesiologists, RBC, Red Blood Cell, Hb, Hemoglobin, Hct, Hematocrit, PLT, Platelet, PT, Prothrombin Time, APTT, Activated Partial Thromboplastin Time, D-D, D-Dimer, MAP, Mean Arterial Pressure

*Student's t test

^†^Mann–Whitney U test

^‡^Chi-squared test

### Intraoperative data

There were no statistically significant differences in surgical time (*P* = 0.643), intraoperative fluid volume (*P* = 0.405), or intraoperative urine output (*P* = 0.317) between the two groups (Table 2).

**Table 2:** Intraoperative data

	NE group (n = 60)	C group (n = 60)	*t*/*Z*	*P* value
Operating duration (min)	138.33 ± 28.04	141.12 ± 37.01	0.464	0.643*
Intraoperative fluid intake (ml/kg)	20.00 (16.15,23.64)	20.14 (17.10,26.15)	− 0.832	0.405†
Intraoperative urine volume (ml/min)	2.26 ± 0.45	2.34 ± 0.42	1.006	0.317*

*Student's t test

^†^Mann–Whitney U test

### Intraoperative blood loss and intraoperative bone-cutting surface bleeding score

**Fig. 2:**
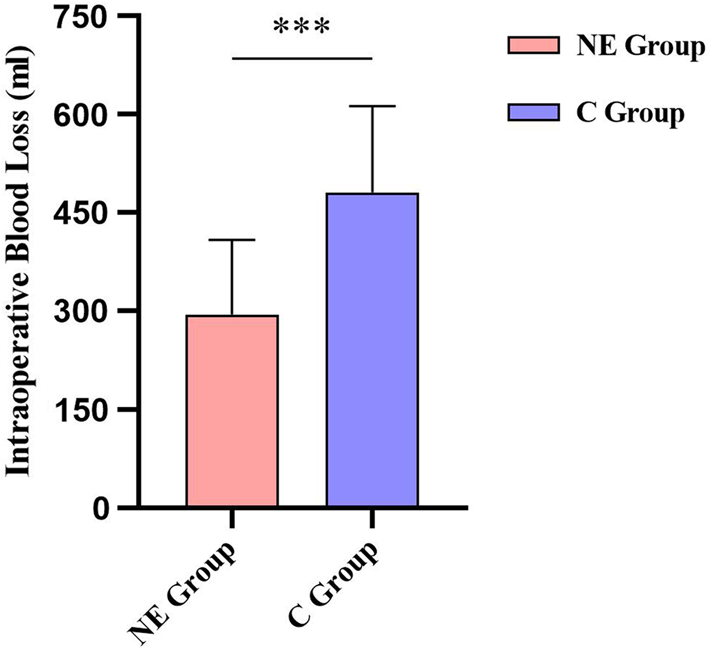
Intraoperative blood loss was compared between the two groups. intraoperative blood loss was significantly reduced in the NE group compared to the C group and the difference was statistically significant. n = 60 for each group and significance was determined using a one-sided independent sample t-test, ***P < 0.001

The average intraoperative blood loss in the NE group and the C group was 294.50 ± 113.71 ml and 480.92 ± 132.05 ml, respectively. Intraoperative blood loss in the NE group was reduced by 38.8% compared to that in the C group, and the difference was statistically significant (*P* < 0.001) (Fig. 2).

**Fig. 3:**
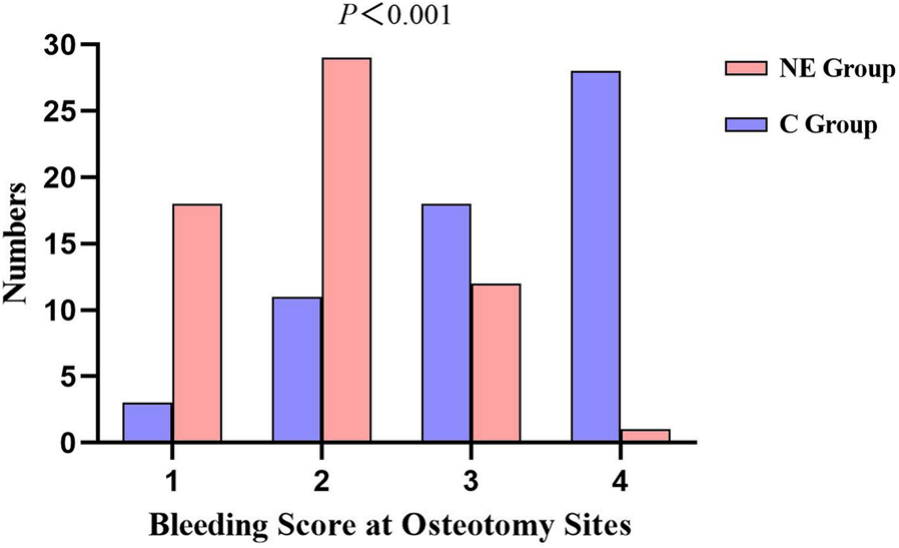
The bleeding score was used to grade the intraoperative bleeding on the distal femoral osteotomy surface in both groups. the NE group had a statistically significant reduction in the median osteotomy surface bleeding score compared to group C. n = 60 for each group, significance determined using the one-sided Mann–Whitney U test, *P* < 0.001

In the NE group, the bone-cutting surface bleeding scores were 1 in 18 cases (30.0%), 2 in 29 cases (48.3%), 3 in 12 cases (20.0%), and 4 in 1 case (1.7%). In group C, the bone-cutting surface bleeding score was 1 in 3 cases (5.0%), 2 in 11 cases (18.3%), 3 in 18 cases (30.0%), and 4 in 28 cases (46.7%). The median bone-cutting surface bleeding scores were 2 (1–2) and 3 (3–4) in the NE and C groups, respectively. The NE group had a significantly reduced bone-cutting surface bleeding score, and the difference was statistically significant (*P* < 0.001) (Fig. 3).

**Fig. 4:**
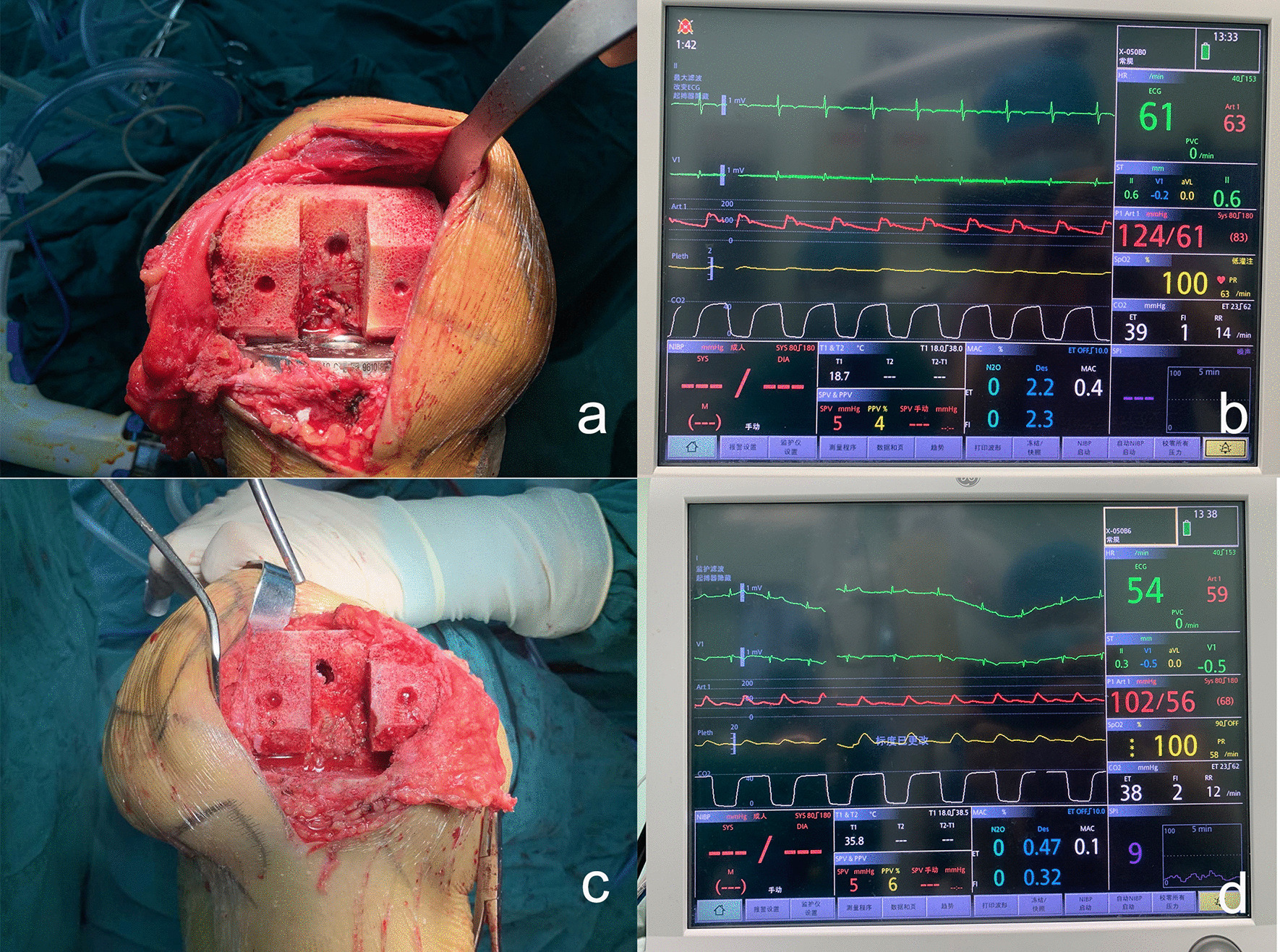
Intraoperative bleeding from the distal femoral osteotomy surface and real-time blood pressure in two typical cases. **a**, **b** A 64-year-old female patient with osteoarthritis of the left knee in group NE had an intraoperative osteotomy surface bleeding score of 2 and a real-time mean arterial pressure of 83 mmHg. **c**, **d** A 62-year-old female patient with osteoarthritis of the right knee in group C had an intraoperative osteotomy surface bleeding score of 4 and a concurrent mean arterial pressure of 68 mmHg

Figure 4 shows the intraoperative bleeding and real-time blood pressure in two typical cases. A typical case in the NE group was that of a 64-year-old female patient diagnosed with "left knee osteoarthritis" before surgery who underwent left total knee replacement. A small dose of norepinephrine was intravenously administered during surgery, and no tourniquet was used. The bone-cutting surface bleeding score was 2 intraoperatively, and the real-time MAP was 83 mmHg (Fig. 4a, b). A typical case in Group C was a 62-year-old female patient diagnosed with "right knee osteoarthritis" before surgery who underwent right total knee replacement. A placebo was administered during surgery and no tourniquets were used. The bone-cutting surface bleeding score was 4 intraoperatively, and the real-time MAP was 68 mmHg (Fig. 4c, d).

### ΔLac before and after administration of norepinephrine

Before and after administration of norepinephrine, the median ΔLac in the NE group was 0.20 (-0.18, 0.70) mmol/L, and in the C group was 0.50 (0, 0.98) mmol/L. The difference in ΔLac before and after administration of norepinephrine between the two groups was not statistically significant (*P* = 0.199) (Table 3).

**Table 3:** Comparison of ΔLac before and after administration of low-dose NE between groups

	NE Group (n = 60)	C Group (n = 60)	*Z*	*P* value
ΔLac (mmol/L)	0.20 (− 0.18,0.70)	0.50 (0,0.98)	− 1.285	0.199*

*Mann–Whitney U test

ΔLac = Difference in arterial lactate levels

### Blood transfusion during hospitalization

During hospitalization, two patients (3.3%) in the NE group and four patients (6.7%) in the C group received blood transfusions. There was no significant difference in transfusion rates between the two groups (*P* = 0.675) (Table 4).

**Table 4:** Comparison of blood transfusion during hospitalization between groups

	NE Group (n = 60)	C Group (n = 60)	*χ*^*2*^	*P* value
Transfusion			0.175	0.675†
Yes	2 (3.3%)	4 (6.7%)		
No	58 (96.7%)	56 (93.3%)		

*Yates's correction for continuity

### Postoperative complications

There was no statistically significant difference in the incidence of postoperative complications within 3 days after surgery between the two groups (*P* = 0.854). None of the patients in the NE group developed myocardial infarction, cerebrovascular accident, oliguria, incision infection, or peripheral limb necrosis after surgery (Table 5).

**Table 5:** Comparison of complications between groups 3 days after surgery

	NE Group (n = 60)	C Group (n = 60)	*χ*^*2*^	*P* value
*Complications*			2.379	0.854*
None	40 (66.7%)	37 (61.7%)		
Deep venous thrombosis	4 (6.7%)	2 (3.3%)		
Intermuscular venous thrombosis	9 (15.0%)	10 (16.7%)		
Swelling	5 (8.3%)	7 (11.7%)		
Pain	1 (1.7%)	3 (5.0%)		
Abnormal healing	1 (1.7%)	1 (1.7%)		

*Fisher's exact test

## Discussion

Tourniquets and controlled hypotension techniques are commonly used in total knee arthroplasty to control bone surface bleeding and maintain a clear surgical field. However, Morelli et al. [10] and Migliorini et al. [9] found that the use of tourniquets often increased the risk of postoperative swelling, pain, and deep vein thrombosis. Jiang et al. [12] reported that controlled hypotension can lead to excessive blood pressure reduction, thereby increasing the risk of organ hypoperfusion complications in elderly patients. Wu et al.'s [19] meta-analysis found that although intravenous tranexamic acid can significantly reduce perioperative and postoperative blood loss in TKA, it cannot reduce intraoperative bleeding or ensure a clear bone-cutting surface. The current concept of bleeding management in TKA has shifted toward the effective reduction of bleeding without increasing the relevant complications, with a high degree of safety. In recent years, studies have reported that flushing bone cutting surfaces with physiological saline solution containing low-dose norepinephrine can significantly reduce perioperative blood loss and transfusion rates in TKA, with no significant complications or adverse reactions [33]. Another study found that intravenous infusion of norepinephrine can counteract the decrease in peripheral vascular tension caused by general anesthesia, constrict resistance vessels, effectively raise blood pressure, and ensure hemodynamic safety [23–25]. This study found that under general anesthesia without tourniquet TKA, intravenous infusion of low-dose norepinephrine can effectively reduce bone-cutting surface bleeding, provide a clearer surgical field without affecting the overall organ perfusion, and increase the transfusion rate during hospitalization, with a high degree of safety, while ensuring that the MAP is not excessively reduced.

We verified the effectiveness of the intravenous infusion of low-dose norepinephrine in reducing bleeding during surgery from two aspects: intraoperative blood loss and the surgeon's visual perception. In this study, the average intraoperative blood loss in the NE group and C group was 294.50 ± 113.71 ml and 480.92 ± 132.05 ml, respectively, with a 38.8% reduction in the NE group compared to the C group. At the same time, the median bone-cutting surface bleeding score in the NE group was 2 (1, 2), while in the C group it was 3 (3, 4), highlighting a significant decrease in the NE group. This indicates that compared with placebo, intravenous infusion of low-dose norepinephrine during TKA without a hemostatic band can significantly reduce intraoperative bleeding and bone-cutting surface bleeding, providing a satisfactory clear bone-cutting surface. De Backer et al. [20] believed that norepinephrine has strong α-adrenergic receptor agonist activity, which can stimulate α-1 adrenergic receptors on peripheral vascular smooth muscle, causing the exposed blood vessels to contract and reduce bleeding. Marchetti et al. [21] and Russell et al. [20] believe that the intravenous infusion of norepinephrine mainly constricts the resistance vessels of small and medium arteries and pre-capillary sphincters, which increases pre-capillary resistance and reduces capillary perfusion pressure, thereby reducing peripheral blood flow. This can explain the effect of the intravenous infusion of low-dose norepinephrine in reducing intraoperative bleeding. In this study, the intraoperative blood loss in both groups was calculated using the hemoglobin balance formula. Gao et al. [34] compared four methods for calculating the intraoperative blood loss in TKA and reported that the hemoglobin balance formula is relatively reliable. The parameters in this formula include hemoglobin concentration before and after surgery, which is easily affected by the intraoperative input and output. When the fluid input is greater than the output, hemoglobin is relatively diluted, which may overestimate the intraoperative blood loss. However, in this study, there was no significant difference in intraoperative fluid replacement and urine output between the two groups. Therefore, we believe that the statistically significant reduction in intraoperative blood loss in the NE group is credible. To the best of our knowledge, this is the first study to quantitatively evaluate the effect of an intravenous infusion of low-dose norepinephrine on intraoperative blood loss and bone-cutting surface bleeding in TKA without a hemostatic band.

Another advantage of using a low dose of norepinephrine via a venous pump is that it can appropriately increase intraoperative blood pressure. Vallée et al. [24] pointed out that anesthesia and analgesics used during general anesthesia reduce sympathetic nerve tension, causing peripheral vasodilation and decreased intraoperative blood pressure. Joosten et al. [25] found that the intravenous infusion of norepinephrine can counteract the decrease in peripheral vascular tension caused by general anesthesia, increase peripheral vascular resistance, and promote the redistribution of blood from peripheral volume vessels to promote circulation. In this study, the NE group demonstrated an increased MAP intraoperatively to 80–90 mmHg through intravenous infusion of low-dose norepinephrine (0.05–0.1 mg/(kg·min)), which was higher than the 65–75 mmHg of the control group. This advantage of reducing intraoperative blood loss and bone surface bleeding scores while maintaining a higher intraoperative MAP is not found in other known intraoperative hemostasis methods.

The greatest risk of intravenous infusion of norepinephrine is excessive vasoconstriction, which leads to inadequate organ perfusion and tissue ischemia. Therefore, the benefits and adverse effects of intravenous infusion of low-dose norepinephrine must be evaluated. To further understand the effect of the intravenous infusion of low-dose norepinephrine on systemic organ perfusion, we compared the differences in arterial blood lactate concentrations between the two groups before and after norepinephrine administration. Lactate is a metabolic product of cells under anaerobic and hypoxic conditions. When the balance between oxygen supply and demand is disrupted, lactate increases due to anaerobic metabolism, and its value directly reflects systemic tissue perfusion and microcirculation [35, 36]. As an increase in the arterial blood lactate concentration is related to tissue ischemia and hypoxia, monitoring changes in the arterial blood lactate concentration can reflect the oxygenation and perfusion of systemic organs and tissues during this stage. In this study, there was no statistically significant difference in the arterial blood lactate concentration before and after drug administration between the two groups, and no organ ischemia-related complications occurred in the NE group postoperatively (including myocardial infarction, cerebrovascular accidents, oliguria, incision infection, and peripheral limb necrosis), indicating that intravenous infusion of low-dose norepinephrine does not affect systemic organ perfusion. This is because the self-regulation of regional blood flow in the human body (including the renal, visceral, cerebral, and coronary vascular beds) is blood pressure-dependent [14]. Organ blood flow can be guaranteed as long as the MAP is maintained at a sufficient value (70–80 mmHg for the kidneys, 50 mmHg for the cerebral and coronary circulation) [16, 37–39]. In this study, the MAP of the NE group was maintained at 80–90 mmHg, which is higher than the threshold of organ self-regulation, so that the organ could maintain relatively sufficient perfusion through its self-regulation mechanism, ensuring the safety of the medication.

Several studies have investigated the optimal dose of intravenously administered norepinephrine. Several animal models of general anesthesia have shown that after appropriate fluid supplementation, intravenous infusion of norepinephrine at 0.3–1 mg/(kg·min) does not affect the blood flow and oxygenation of visceral organs such as the heart, kidneys, liver, and intestines [23, 40–42]. A study in breast cancer surgery indicated that intravenous infusion of norepinephrine at 0.1 mg/(kg·min) had no effect on tissue microcirculation [43]. Another randomized controlled study on radical cystectomy indicated that the intravenous infusion of norepinephrine at 0.06 mg/(kg·min) did not increase blood lactate levels or decrease central venous saturation.. As there is currently a lack of reports on the safety of using higher doses of norepinephrine intravenously during human general anesthesia, we chose 0.05–0.1 mg/(kg·min) as the target dose of norepinephrine in this study, ensuring experimental efficacy and safety.

There was no significant difference in transfusion rates between the two patient groups during hospitalization; however, the overall transfusion rate in our study population was very low (5%), with two patients (3.3%) in the NE group and four patients (6.7%) in the C group receiving transfusions. In this study, both groups of patients received a dose of tranexamic acid (15 mg/kg) via intravenous infusion before and 3 h after the surgery. Although tranexamic acid cannot reduce intraoperative blood loss, numerous randomized controlled trials have shown that intravenous infusion of tranexamic acid without a tourniquet during TKA can effectively and safely reduce postoperative bleeding and transfusion requirements [18, 19, 44]. The reduced postoperative bleeding may have contributed to the overall low transfusion rate during hospitalization. In addition, the transfusion criteria in our study (hemoglobin concentration less than 70 g/L) were relatively strict, potentially concealing certain transfusion needs. Based on our results, we believe that the venous infusion of norepinephrine, without inferiority to blood transfusion, is associated with the use of intravenous tranexamic acid during surgery.

This study was conducted in patients receiving routine general anesthesia, which is the standard anesthetic method for TKA at our center. While certain studies have indicated that general anesthesia results in greater blood loss when compared to spinal anesthesia [45], the growing preference for general anesthesia in TKA can be attributed to advancements in anesthesia techniques, medications, and the enhancement of patient comfort during the anesthesia procedure.. Harsten et al. [27] and Neal-Smith et al. [45] confirmed that surgery under general anesthesia is more effective and can reduce patient anxiety during the perioperative period, leading to overall better postoperative outcomes. Therefore, the significant results of this study are limited to TKA under general anesthesia, and we cannot draw additional conclusions about other anesthesia methods.

This study has several limitations. First, some studies have shown that norepinephrine can promote platelet release and activation by activating α-2 and β-2 adrenergic receptors, which stimulates the release of various coagulation factors to achieve hemostasis [46–49]. Previous randomized controlled studies have shown that low concentrations of norepinephrine (approximately 4.2 nmol/L) in the blood circulation are sufficient to dose-dependently activate platelets, enhance platelet aggregation and secretion, and increase the expression of platelet surface fibrinogen receptor [50–52]. Consequently, we cannot disregard the potential impact of the procoagulant effect of norepinephrine on intraoperative bleeding. Owing to logistical reasons, we were unable to directly measure intraoperative coagulation indicators, which represents a significant limitation of this study. Second, the difference in arterial blood lactate concentrations before and after norepinephrine administration in this study only reflects the overall oxygenation and perfusion of systemic organs and tissues. However, the blood flow and perfusion of various organs during surgery are extremely complex and variable. Therefore, we need to conduct independent evaluations of the perfusion of important organs, such as the heart, brain, and kidneys, by measuring indicators such as cardiac troponin, local cerebral oxygen saturation, blood creatinine, blood urea nitrogen, and glomerular filtration rate. This will be the next research direction.

In summary, venous infusion of low-dose norepinephrine can effectively reduce intraoperative bleeding and provide a clearer bone-cutting surface while ensuring that the MAP is not low. Simultaneously, venous infusion of low-dose norepinephrine did not affect the blood lactate concentration indicators of systemic organ perfusion during surgery and did not increase the transfusion rate during hospitalization, indicating high safety. These are the unique advantages of venous infusion of low-dose norepinephrine that other known intraoperative hemostasis methods do not have. This study proposes a novel and feasible approach for optimizing intraoperative bleeding management and increasing surgical safety.

## Conclusion

During general anesthesia for total knee replacement surgery using a tourniquet, intravenous infusion of low-dose norepinephrine can safely and effectively reduce intraoperative blood loss. This intervention also maintains MAP, thereby ensuring a clearer bone-cutting surface.

## Data Availability

Data supporting the findings of this study are available upon request from the corresponding authors.

## Abbreviations

NE: Norepinephrine
TKA: Tourniquet total knee arthroplasty
MAP: Mean arterial pressure
POD1: Postoperative day 1
IPC: Intermittent pneumatic compression
BMI: Body mass index
RBC: Red blood cell count
Hb: Hemoglobin
Hct: Hematocrit
PLT: Platelet count
PT: Prothrombin time
APTT: Activated partial thromboplastin time
SD: Standard deviation
IQR: Interquartile ranges
